# Comparison of two different mechanical esophagogastric anastomosis in esophageal cancer patients: a meta-analysis

**DOI:** 10.1186/s13019-015-0271-4

**Published:** 2015-05-08

**Authors:** Dong Zhou, Quan-Xing Liu, Xu-Feng Deng, Jia-Xin Min, Ji-Gang Dai

**Affiliations:** Department of Thoracic Surgery, Xinqiao Hospital, Third Military Medical University, Chongqing, 400037 China

**Keywords:** Linear, Circular, Anastomosis, Leakage, Strictures, Meta-analysis

## Abstract

**Objective:**

In this meta-analysis, we conducted a pooled analysis of clinical studies comparing Linear Stapled (LS) versus Circular Stapled (CS) esophagogastric anastomosis for esophageal cancer.

**Methods:**

According to the recommendations of the Cochrane Collaboration, we established a rigorous study protocol. We performed a systematic electronic search of the PubMed, Embase, Cochrane Library, Web of Science, and Chinese Biomedical databases as well as Chinese scientific journals to identify articles to include in our meta-analysis. The primary outcomes compared were anastomotic leak, anastomotic stricture and 3-month mortality.

**Results:**

Five controlled trials comprising 840 patients (523 LS vs. 317 CS) were included. Primary outcomes revealed a statistically significant decrease in anastomotic strictures [risk ratio (RR): 0.26, 95 % confidence interval (CI): 0.11–0.60, P = 0.002] compared with linear stapled anastomosis. However, there were no significant differences between the two groups with respect to anastomotic leakage [risk ratio (RR): 0.80, 95 % confidence interval (CI): 0.40–1.58, P = 0.52] and 3-month mortality [risk ratio (RR): 0.94, 95 % confidence interval (CI): 0.47–1.87, P = 0.85].

**Conclusion:**

There were no statistical differences in the rate of 3-month mortality or anastomotic leakage between the two groups. However, the LS method contributed to a reduced rate of anastomotic strictures. This meta-analysis may offer some specific suggestions for esophagogastric anastomosis.

## Background

Esophageal carcinoma is a multifaceted and complex disease of rapidly rising incidence that exerts an increasing social and financial burden on global healthcare systems [1–4]. Currently, esophagectomy is the gold standard treatment for esophageal carcinoma. The stomach is the most common substitute after esophagectomy for patients with esophageal carcinoma [5, 6]. However, the major complications after esophagectomy, such asanastomotic leakage, anastomotic stricture, and gastroesophageal reflux, are frequently encountered, and these complications can compromise patient quality of life and maybe life-threatening. Therefore, finding effective methods to promote healing of an anastomosis and to prevent anastomotic leakage or stricture formation remains a problem in esophageal surgery [7, 8]. Currently, various surgical techniques are used for the construction of the esophagectomy to produce better outcomes, such as circular stapled anastomosis, linear stapled anastomosis and hand-sewn anastomosis.

The circular stapled anastomosis has become increasingly popular since the 1990s. The linear stapled anastomosis was first described by Collard et al [9] in 1998 and involves side-to-side esophagogastric anastomosis using a small linear stapler; the procedure was later modified by Orringer et al [10]. In the procedure the linear stapled structure comprises two double-staggered rows of staples, and the tissue can be cut between the double rows simultaneously. In the linear stapler suture technique, the two forks of an Endo-GIA stapler (US Surgical Corp, Norwalk, CT) are placed across the two opposing walls with the anvil in the gastric lumen and the cartridge of staples in the esophageal lumen [11, 12]. After approximation of the two forks, the trigger of the stapler is squeezed to allow forward displacement of the knife and the delivery of three rows of staples on each side. After the two forks have been separated, the stapler is removed, and the two stapled wound edges retract laterally in response to the action of the intramural musculature. The medial slit thus becomes a V-shaped opening between the two lumina. The two posterior walls realign themselves by exerting gentle downward traction on the transplant. The anterior walls are sutured to each other using a single-layer running suture technique similar to that used in hand-sewn anastomoses.

All methods have their own specialised advantages and disadvantages, and important complications due to anastomotic leakage and stricture formation are well known. Several studies have been performed to compare the traditional hand-sewn anastomosis method to the modern mechanical stapled anastomosis method. However, the evidence that comparing LS with CS anastomosis in esophagectomy has only been reported in a few small trials [13–17]. Moreover, there has been no meta-analysis comparing LS anastomosis with CS anastomosis for esophageal cancer. Thus, we conducted a meta-analysis that compared LS with CS methods for esophagogastric anastomosis after esophagectomy and observed the contribution of each method to the incidence of anastomotic leakages, anastomotic strictures and 3-month mortality. This is the first meta-analysis comparing LS to CS esophagogastric anastomosis for esophageal cancer. Through this analysis, we aim to gain a greater understanding of the collective impact of these parameters and their contribution to anastomosis failure.

## Materials and methods

We performed a systematic electronic search of the PubMed, Embase, Cochrane Library, Web of Science, and Chinese Biomedical databases as well as Chinese scientific journals to identify articles for inclusion in our meta-analysis. Thesearch terms ‘esophagectomy’, ‘anastomosis’, ‘linear’, ‘circular’, ‘stapled’ and ‘gastric’ and the MeSH headings ‘anastomosis’ (MeSH), ‘linear’ (MeSH), ‘circular’ (MeSH) ‘stapled’(MeSH) and ‘esophagectomy’ (MeSH) were used in combination with the Boolean operators AND or OR. The electronic search was supplemented by a hand-search of published abstracts from the annual meetings of relevant surgical societies. In reference searches, lists of trials selected from electronic searches were scanned to identify further relevant trials.

Abstracts of the citations identified by the search were then scrutinized by two observers to determine eligibility for inclusion in the meta-analysis. Studies were included if they met each of the following criteria: comparative studies and separation into groups based on the use of linear stapled and circular stapled anastomosis for esophagectomy surgery. Our search identified 5 studies that met our criteria in the meta-analysis [13–17]. The data extracted from each article included the study design, number of subjects, male/female ratio, mean age of subjects, and any preoperative interventions performed.

The primary outcome measures for the meta-analysis were anastomotic leakage and 3-month mortality. The secondary outcome measures for the meta-analysis was anastomotic strictures (developing within 6 months of operation requiring endoscopy). Data from eligible trials were entered into a computerized spreadsheet for analysis. The quality of each trial was assessed using the Jadad scoring system. We performed the meta-analysis using the RevMan5.1.9 software (provided by the Cochrane Collaboration, Oxford, UK) for the controlled studies. The relative risk (RR) was calculated using 95 % confidence intervals (CI). We used the *χ*2 statistic to assess statistical heterogeneity and the Higgins I^2^ statistic to determine the percentage of total variations across studies due to heterogeneity. If the I^2^ statistic was ≤50 %, the fixed effect model was used to pool studies; otherwise, the random effects model was used.

## Results

### Study characteristics

**Fig. 1:**
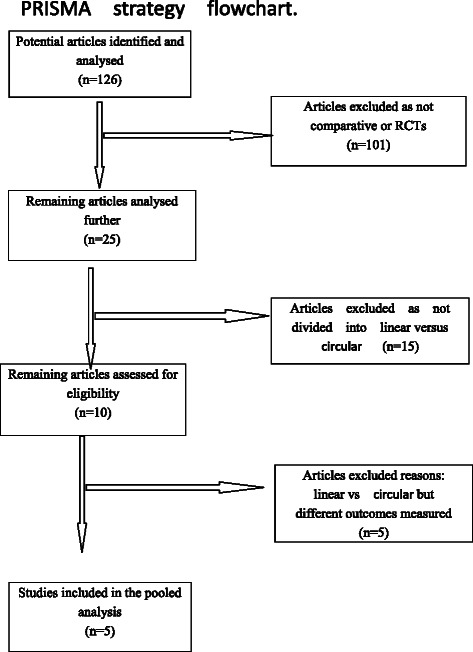
Flow chart of the literature search according to the PRISMA statement

After screening, five studies comprising 840 patients (523 Linear vs. 317 Circular) were included. Fig. 1 presents an overview of the literature search performed for the systematic review and meta-analysis according to the PRISMA statement. Patient demographic data for each trial are represented in Table 1.

**Table 1:** Characteristics of the included trials

Author	Publication year	Anastomotic method	No. of patients	Male/female ratio	Age
Yoshiyuki F	2005	LS	12	Details unknown	-
		CS	8		-
Shanda H. B	2007	LS	44	Details unknown	61.0 ± 9.0
		CS	147		62.0 ± 12.0
Qi-Rong Xu	2010	LS	162	143 /23	60.2 ± 8.4
		CS	67	61/7	61.3 ± 7.6
Wen-Ping Wang	2013	LS	45	41/4	59.7 ± 7.4
		CS	47	41/6	61.4 ± 7.7
Theolyn N. P	2013	LS	260	Details unknown	64.0
		CS	48		64.0

### Gastroesophageal anastomotic strictures

**Fig. 2:**
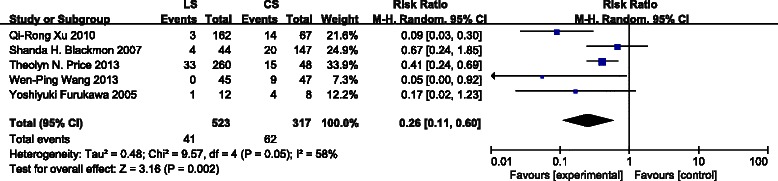
Forest plot for anastomotic strictures. Five studies were included

All 5 studies reported the incidence of gastroesophageal anastomotic strictures following circular stapled versus linear stapled anastomosis. The use of a LS method decreased the incidence of developing anastomotic strictures relative tithe CS method (Fig. 2) [risk ratio (RR): 0.26, 95 % confidence interval (CI): 0.11–0.60,P = 0.002]. Heterogeneity was found to be significant [I^2^ = 58 %,*χ*^2^ = 9.57 (df = 4), P = 0.05].

### Gastroesophageal anastomotic leakage

**Fig. 3:**
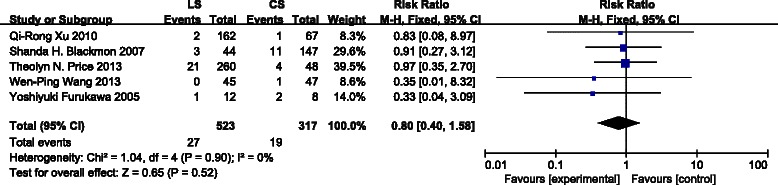
Forest plot for anastomotic leakage. Five studies were included

All 5 studies reported the incidence of gastroesophageal anastomotic leakage. There was no statistical difference between the groups with respect to anastomotic leakage (Fig. 3) [risk ratio (RR): 0.80, 95 % confidence interval (CI): 0.40–1.58, P = 0.52]. Statistical heterogeneity was not detected [I^2^ = 0 %,*χ*^2^ = 1.04 (df = 4), P = 0.90].

### Mortality within 3-months of surgery

**Fig. 4:**
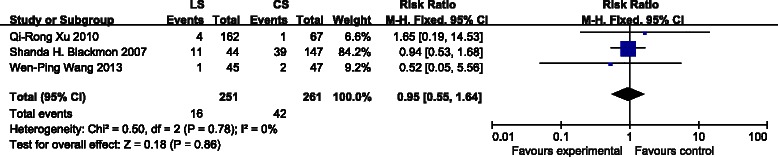
Forest plot for 3-month mortality. Three studies were included

Only 3 studies reported the 3-month mortality outcomes. There were no statistical differences in mortality between the LS and the CS groups (Fig. 4) [RR: 0.95, 95 % confidence interval (CI): 0.55–1.64; P = 0.86]. Statistical heterogeneity was not detected [I^2^ = 0 %, *χ*2 = 0.50, (df = 2), P = 0.78].

## Discussion

In the early days, the success rate of hand-sewn anastomosis was extremely low. As the technology progressed, the anastomosis technique improved. Nevertheless, the evidence comparing LS with CS anastomosis in esophagectomy has only been reported in a few small trials. The first article on this topic was published in 2005 [17]. The authors concluded that the advantages of the linear stapled approach for gastroesophageal anastomosis include less anastomotic leakage and fewer strictures relative to other anastomosis methods. However, the authors did not attempt to produce a meta-analysis that could have helped produce a statistically sound argument on this topic. In our present meta-analysis, we have attempted to review the published controlled trials on this topic to date to gain a pooled analysis and a consensus of the best anastomotic practice.

Our review provides a comprehensive summation of the current literature describing the outcomes with LS or CS anastomosis after esophagectomy. For our study, attempts were made wherever possible to closely follow the recommendations presented by the Cochrane Collaboration [14]. We performed a rigorous study protocol and searched several electronic databases without restrictions on language. Our search identified only 5 studies that met our criteria in the meta-analysis [7–11]. However, we also believe that one of the significant merits of our meta-analysis is that we performed the analyses by the prespecified protocol. Thus, this revisited meta-analysis might provide answers to surgeons’ concerns of statistical power and superior quality analyses.

Our meta-analysis revealed 3 significant findings. First, there were no statistical differences in the incidence of developing anastomotic leakage, the most severe complication, between the LS and the CS group. Second, the use of LS anastomosis contributed to reducing the rate of anastomotic strictures compared with CS anastomosis. Statistical heterogeneity was detected in the rate of anastomotic strictures, possibly due to 2 outliers. Although this analysis reveals an association between decreased rate of anastomotic stricture and the use of the LS method, it was not possible to fully quantify this relationship with an objective measurement of inner anastomotic diameter. A further factor that can influence gastroesophageal diameter following LS anastomosis is the depth of stapler introduction. Finally, we also conducted an analysis using a fixed-effects model concerning mortality. There were no statistical differences in the risk of 3-month mortality between the LS and the CS groups.

The development of anastomotic strictures at the level of the gastroesophageal anastomosis is a well-recognized complication, the incidence of which ranges from 13.60 % to 31.25 % [14, 16]. The etiology of stricture formation is likely multifactorial and includes exposure to excessive gastric acid, subclinical leaks, the degree of tension and local ischemia on the anastomosis [18, 19].

Our meta-analysis has limitations because of the sample size. The fact that we could not identify the effect modifiers may be attributable to the low statistical power. There also remained unexplained heterogeneity in the study of anastomotic stricture. Moreover, the number of trials and the number of patients was relatively small. An additional limitation is the potential interaction and crossover between several technical factors evaluated, which was controlled in this pooled analysis. Future trials should include standard measures to allow objective and comparable assessment of the outcomes. Standardization was lacking in the reporting of treatment outcomes, trial lengths, and the proportion of the recruited sample that was followed. Several trials failed to accurately present such information. Disease recurrence may also be underestimated because some studies used telephone contact or questionnaire-based follow-up. Therefore, it is possible that a small number of patients with macroscopic but symptomatic recurrence may not be detected using such assessment techniques.

## Conclusion

In conclusion, there were no statistical differences in the rate of 3-month mortality or anastomotic leakage between linear stapled (LS) and circular stapled (CS) esophagogastric anastomosis in our meta-analysis. However, a decreased rate of stricture formation was observed in the LS group, which is likely the result of an independent factor specific for the mechanical anastomosis and not related to the healing of a previous anastomotic leakage. Therefore, our meta-analysis suggests that LS anastomosis should remain the first-line approach because of the substantial benefits of using the LS technique relative to the CS technique with respect to rates of stricture formation. Although cost was not formally assessed in this analysis, it may be inferred from these benefits that there is a reduced cost associated with LS anastomosis.
